# Therapeutic Endoscopic Ultrasound in Biliopancreatic Disease

**DOI:** 10.3390/jcm15082848

**Published:** 2026-04-09

**Authors:** Aurelio Mauro, Carlotta Crisciotti, Giulio Massetti, Daniele Alfieri, Stefano Mazza, Davide Scalvini, Alessandro Cappellini, Guglielmo Aprile, Gianmaria La Rosa, Francesca Torello Viera, Letizia Veronese, Marco Bardone, Andrea Anderloni

**Affiliations:** 1 Gastroenterology and Digestive Endoscopy Unit, Fondazione I.R.C.C.S. Policlinico San Matteo, 27100 Pavia, Italy; 2 Department of Internal Medicine and Therapeutics, University of Pavia, 27100 Pavia, Italy

**Keywords:** therapeutic endoscopic ultrasound, benign biliary disease, malignant biliary obstruction, EUS-gallbladder drainage, EUS-choledochoduodenostomy, EUS-hepaticogastrostomy, EUS-gastroenterostomy, EUS-radiofrequency ablation

## Abstract

Therapeutic endoscopic ultrasound (t-EUS) has transformed the management of biliopancreatic diseases by enabling minimally invasive access and intervention through the gastrointestinal wall. This narrative review summarizes current indications and evolving roles of t-EUS in benign and malignant biliary disease, with a focus on the different modalities of transmural drainage, EUS-guided gastroenterostomy (EUS-GE), and EUS-guided radiofrequency ablation (EUS-RFA). In benign settings, EUS-gallbladder drainage (EUS-GBD) has emerged as a minimally invasive alternative to percutaneous cholecystostomy for high-risk patients with acute cholecystitis, offering internal drainage with fewer tube-related adverse events. In malignant biliary obstruction, transmural drainages are consolidated alternatives of endoscopic retrograde cholangiopancreatography (ERCP) as first-line or rescue strategies, providing durable internal biliary drainage, avoiding post-ERCP pancreatitis without deteriorating quality of life. In surgically altered anatomy, t-EUS overcomes the limitations of enteroscopy-assisted ERCP by creating direct access routes to the biliary tree or pancreatic duct. EUS-guided pancreatic duct drainage offers a rescue or primary approach in benign strictures, anastomotic stenosis, and disconnected duct syndrome. EUS-GE has rapidly become a preferred modality for palliation of gastric outlet obstruction in pancreatic cancer, while EUS-RFA provides a platform for locoregional therapy in selected cases of pancreatic neuroendocrine tumors, adenocarcinoma, and pancreatic cystic neoplasms. Collectively, these applications position t-EUS as a central tool in the multidisciplinary management of complex biliopancreatic disease, with ongoing innovations expected to further expand its indications and safety and to refine patient selection and training pathways.

## 1. Introduction

Endoscopic ultrasound (EUS) has undergone a transformative evolution since its introduction as a diagnostic imaging modality for gastrointestinal and adjacent structures. The integration of high-frequency ultrasound with endoscopy enabled detailed visualization and, subsequently, tissue acquisition, rapidly establishing EUS as a cornerstone in the diagnosis of benign and malignant pancreatobiliary diseases [1]. The advent of linear echoendoscopes and the development of dedicated accessories marked a defining moment, allowing for real-time guidance of therapeutic interventions such as drainage, ablation, and anastomosis [2].

Therapeutic EUS (t-EUS) now encompasses a spectrum of minimally invasive procedures, including biliary drainage as an alternative of endoscopic retrograde cholangiopancreatography (ERCP), management of peripancreatic fluid collections, and palliation of malignant obstruction [3]. The rationale for its adoption is driven by the need for effective alternatives to surgical and radiologic approaches, offering reduced morbidity, shorter hospital stays, and improved quality of life, particularly in oncologic and high-risk populations [3]. Recent evidence has positioned EUS-guided interventions as first-line and rescue therapies in complex biliopancreatic scenarios, including altered anatomy [4].

This narrative review will address the current landscape and future directions of t-EUS, with dedicated subsections on biliary drainage (benign, malignant, altered anatomy), pancreatic drainage, EUS-guided gastroenterostomy (EUS-GE), and EUS-guided radiofrequency ablation (EUS-RFA).

## 2. Biliary System Drainage

### 2.1. Benign Disease

Despite ERCP being the gold standard of treatment in the management of benign biliary pathologies, such as stones and benign strictures, it may fail in up to 10% of cases even when performed by expert endoscopists [5]. Where ERCP fails, t-EUS techniques can be applied to optimally manage these cases and especially to offer a same-session solution to biliary access [6].

#### 2.1.1. EUS Rendezvouz

Among the t-EUS techniques applied to the management of benign diseases, the EUS-guided rendezvous (EUS-RV) approach is a key salvage strategy when conventional ERCP fails. This procedure involves EUS-guided puncture of the biliary system, advancement of a guidewire through the papilla or biliary-enteric anastomosis, and subsequent wire-guided ERCP to achieve biliary access and intervention. Multiple passages are needed and high expertise is required to finalize the procedure. Primary indications for EUS-RV in benign diseases are situations of difficult or impossible biliary cannulation, for example, in cases of an intradiverticular papilla, surgically altered anatomy (SAA), chronic pancreatitis with papillary stenosis, or other benign biliary strictures where standard biliary access with ERCP cannot be achieved [7]. In SAA cases though, it is essential that the papilla be within reach of the endoscope (e.g., Billroth II, short afferent limb). In these contexts, EUS-RV serves as a minimally invasive rescue option to establish biliary access without resorting to conventional alternatives such as percutaneous-RV that imply a second-session procedure with consequent prolonged hospitalization time and reduced quality of life related to the percutaneous access [8]. By contrast, EUS-RV achieves internal drainage via the natural biliary outflow tract, thus preserving normal anatomy and avoiding external medical devices [8]. This is a key conceptual advantage in benign disease, where long-term biliary tract integrity is desirable and the creation of a permanent transmural fistula may be unnecessary.

American guidelines state that in patients with biliary obstruction and failed ERCP, the EUS-RV procedure is preferred over other EUS-guided transmural drainage techniques [such as EUS-guided choledochoduodenostomy (EUS-CDS) or EUS-guided hepaticogastrostomy (EUS-HGS)] in cases of suspected benign disease after multidisciplinary consultation [9].

Recent meta-analyses demonstrate that EUS-RV achieves high technical and clinical success rates in benign biliary disease [10,11,12,13]. Adverse event (AE) rates are reported between 10 and 19%, with most complications being mild to moderate (both EUS and subsequent ERCP combined). Major AEs are less frequent but underscore the need for procedural expertise [10,11,12]. Such complications include bile leak, biliary peritonitis, post-ERCP pancreatitis, and hemorrhagic and infectious complications [14,15]. A recently published report highlights the technical feasibility of performing EUS-GBD and EUS-RV in a single session for patients with acute cholecystitis and concurrent biliary stones when conventional ERCP is not possible due to anatomical challenges, particularly if patients are high-risk surgical candidates [15].

#### 2.1.2. Gallbladder Drainage

In high-risk surgical patients with acute cholecystitis, EUS-guided gallbladder drainage (GBD) with electrocautery-enhanced lumen-apposing metal stents (EC-LAMSs) has matured into a definitive alternative to percutaneous transhepatic gallbladder drainage (PT-GBD) [16,17]. Frailty, advanced age, and existing comorbidities define a high-risk surgical candidate, constituting the population gaining the most benefit from this endoscopic approach [18]. EUS-guided GBD procedure can be performed via the transgastric or the transduodenal route, the latter seeming to be the preferred approach as the stent is less impacted by gastric content and long-term complications; however, duodenal drainage is not always technically feasible, and the gastric route is a valid alternative especially if a co-axial double pigtail stent is used [19].

European and American guidelines favor EUS-GBD over the percutaneous route owing to the lower rates of complications and need for re-intervention in this type of population [16,20]. Randomized and controlled comparative studies consistently show EUS-GBD achieves at least an equivalent clinical resolution with advantages beneficial to patients when performed with EC-LAMSs: no external catheter, fewer tube-related issues, and fewer gallbladder-related reinterventions. Network and conventional meta-analyses corroborate these advantages [21] and propensity-matched comparisons with interval laparoscopic cholecystectomy highlight that, in high-surgical-risk patients, EUS-GBD could be at least comparable to surgery, but randomized trials are needed [22]. Early AEs include bile leak, cholangitis, hemorrhage, and duodenal perforation (if performed via the transduodenal route), whilst stent occlusion and migration are the most common late AEs [14].

Results from a prospective study post-EUS-GBD suggested no increase in the risk of AEs in patients with long-term LAMS indwell, concluding that LAMS misdeployment was the strongest risk factor for AEs [23]. Notably, many AEs are usually treated conservatively or endoscopically [24]. Further studies are needed to assess long-term outcomes, especially in comparison with surgery to strengthen the role of EUS-guided GBD for treatment of acute cholecystitis in frail patients. The procedure requires dedicated training, with the most recent data suggesting that at least 23–27 supervised cases are needed to achieve competence [25]. In centers without EUS expertise, PT-GBD remains a valid and accessible alternative.

### 2.2. Malignant Disease

Malignant biliary obstructions (MBOs), often due to pancreatic head adenocarcinomas or distal cholangiocarcinomas, are traditionally managed by ERCP with stent placement as first-line therapy [26]. Nevertheless, ERCP may fail and carries a noteworthy risk of AEs, such as post-ERCP pancreatitis, which rarely results in death [27].

#### 2.2.1. Choledochoduodenostomy

EUS-guided CDS has emerged as an effective alternative to achieve biliary drainage in MBO, earning validation as a second-line treatment after failed ERCP [16,28,29] usually related to infiltration of the duodenopapillary area. EUS-CDS implies using the transduodenal route to puncture the dilated common bile duct (CBD) from the duodenal bulb, followed by placement of a LAMS to create choledochoduodenal anastomosis.

In recent years, the application of EUS-CDS progressively anticipated the execution of ERCP as a first-line approach or as a rescue strategy in difficult biliary cannulation. In such cases, performing early EUS-CDS before the application of advanced cannulation techniques (e.g., needle-knife precut, double guidewire or transpancreatic sphincterotomy) may be as effective as first-line ERCP and may reduce the risk of AEs, especially post-ERCP pancreatitis [30]. In the setting of first-line approaches, early randomized controlled trials showed similar success rates to ERCP with lower rates of pancreatitis and reinterventions [29,31,32]. Recent multicenter trials using EC-LAMSs confirmed non-inferior or superior technical success, comparable clinical outcomes, and fewer complications [28,33,34]. Meta-analyses further reinforce that EUS-CDS matches ERCP in efficacy, virtually eliminates risk of post-procedure pancreatitis, and lowers stent dysfunction rates, particularly when LAMSs are used [26].

EUS-CDS can be performed during the same session of EUS-FNB, allowing tissue acquisition and stenting in one session, offering a logistical advantage over the ERCP approach [33]. Another emerging indication is preoperative drainage in borderline or locally advanced pancreatic cancers undergoing neoadjuvant therapy [34,35]. Effective biliary drainage is often required before chemotherapy or surgery, and EUS-CDS offers internal drainage without the papillary manipulation that could induce pancreatitis or cholangitis.

These findings support EUS-CDS as a viable strategy in expert centers, including in patients receiving neoadjuvant therapy. Critically, however, EUS-CDS carries a learning curve that is estimated at approximately 30 cases to achieve consistent technical success [36], and its adoption outside tertiary centers remains limited by equipment availability and training requirements. Additionally, not all patients with malignant distal biliary obstruction are suitable candidates: EUS-CDS requires a dilated common bile duct (typically ≥15 mm), absence of coagulopathy, and a bile duct accessible from the duodenal bulb, which may be compromised by duodenal infiltration or distortion [34]. These anatomical and patient-related selection criteria must be carefully assessed before proceeding, and the decision should ideally involve multidisciplinary input.

The AEs tied to EUS-CDS can be challenging to manage in patients with advanced malignancy. A prospective study highlighted how EUS-CDS may result in a higher risk of biliary dysfunctions, cholangitis in particular, especially in patients with duodenal obstruction [37]. To prevent stent obstruction related to food impaction, a recent RCT proposed the use of a coaxial double pigtail plastic stent inside the LAMS, as already used in EUS-PFC [38]. These findings justify the need for more long-term data on large cohorts of patients.

#### 2.2.2. Hepaticogastrostomy

Another method to obviate MBO and achieve effective biliary drainage is by creating a diversion from the intrahepatic bile ducts to the digestive lumen under EUS guidance, usually between the left hepatic system and the stomach, otherwise known as an EUS-guided hepaticogastrostomy. EUS-HGS is one of the most challenging t-EUS procedures: it requires multiple intraprocedural accessory changes with the need for careful, simultaneous control and execution of consecutive steps under endoscopic, ultrasonographic and fluoroscopic coordination [39]. Currently, its indications include palliation of both distal and proximal biliary obstruction. In case of distal MBO, HGS is usually applied when ERCP fails and the other t-EUS techniques are not feasible (e.g., duodenal obstruction, surgically altered anatomy [16,40]). Despite this, EUS-CDS and EUS-HGS demonstrated comparable safety and efficacy for biliary drainage after failed ERCP in distal MBO in clinical trials [39,41,42,43,44]. However, complications that may occur during or after HGS could be more challenging to manage compared to EUS-CDS, especially considering the setting of malignant palliation. Recent studies support the use of HGS as a first option when there is a concomitant biliary and duodenal obstruction or in SAA with long afferent limb such as in cases of Roux-en-Y reconstructions [37,40,45].

In cases of proximal MBO, such as complex hilar strictures, EUS-HGS may be considered as a stand-alone approach after failed ERCP, or even in combination with ERCP [16].

Combination approaches may further optimize drainage in complex hilar cases, though they may increase the risk of pancreatitis [46,47,48].

From a clinical decision-making perspective, EUS-HGS is technically the most demanding of all t-EUS biliary drainage procedures and should be reserved for centers performing a high volume of EUS-guided interventions (generally defined as more than four procedures per year based on meta-regression data) [43]. The most common AEs of EUS-HGS are cholangitis, sepsis, bleeding, stent migration, stent occlusion, peritonitis, and bile leak, the latter two being the most important early complications [14,24,43,49]. Risk factors for these adverse events include technical and patient-related variables. A short distance between the hepatic parenchyma and the gastric wall is a strong independent risk factor for bile peritonitis, as it increases the likelihood of bile leakage into the peritoneal cavity [50]. Multiple punctures, prolonged procedure time, and the presence of acute cholangitis at the time of the procedure also increase the risk of bile leak and peritonitis [50]. Procedures perfomed in less experienced centers and the use of non-dedicated stents are associated with higher overall adverse event rates [43]. The steep learning curve and the severity of potential complications mean that EUS-HGS should not be attempted outside experienced hands.

**Figure 1:**
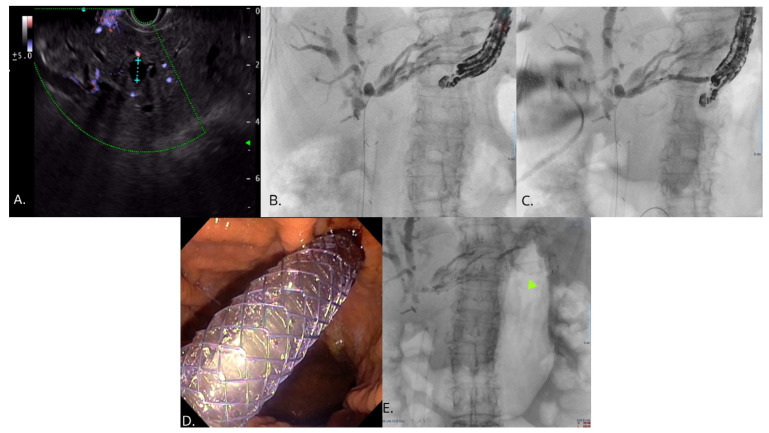
Hepaticogastrostomy steps. (**A**) Ultrasound view of the liver parenchyma. The application of color doppler allows the recognition of the dilated intrahepatic biliary ducts (within the caliper). (**B**) Under EUS guidance, a dilated intrahepatic biliary duct in segment III is accessed by puncture through the gastric wall, followed by contrast injection to visualize the biliary tree. A guidewire is then advanced under fluoroscopic view. A duodenal SEMS was previously placed to overcome a neoplastic stenosis. (**C**) The newly created fistulous tract is dilated with a dilation balloon. (**D**) A partially covered metallic stent is then released, connecting the right intrahepatic biliary system with the gastric lumen. (**E**) Final fluoroscopic view of the stent, as highlighted by the green arrow.

Innovations in stent design have focused on dedicated partially covered self-expandable metal stents (PC-SEMS) with anti-migratory features, such as anchoring hooks, flared ends, and thin delivery systems, all features that have contributed to lower rates of AEs [43,51]. Figure 1 illustrates the steps required to perform EUS-HGS.

#### 2.2.3. Gallbladder Drainage

Another option in case of failed ERCP is EUS-GBD [19]. It also represents a rescue strategy to achieve biliary decompression in cases where EUS-CDS has proved insufficient or unfeasible [52].

This procedure demonstrates a high technical and clinical success rate in the management of MBO, particularly in patients with a patent cystic duct and no prior cholecystectomy, criteria amongst the indications to undergo this procedure [53,54,55,56]. Whilst EUS-GBD is frequently used as a salvage strategy when EUS-CDS is not feasible (usually when the CBD is not sufficiently dilated or there is a duodenal malignant infiltration), it may also be considered as a primary approach when cystic duct patency is confirmed [54]. EUS-GBD is associated with a significantly lower rate of 6-month reintervention and stent-related complications compared to EUS-CDS, while overall adverse event rates and all-cause mortality are similar between the two techniques. These findings are supported by an international multicenter trial, which found no significant differences in technical or clinical success, serious adverse events, or overall survival between EUS-GBD and EUS-CDS as first-line therapies for MBO [53,54,55]. However, cystic duct patency is not always easily evaluated, and jaundice reduction occurs more slowly than EUS-CDS.

Safety profiles for EUS-GBD are favorable, and most complications are mild to moderate in severity and are easy to treat conservatively and endoscopically [24]. The most common adverse events include stent migration, stent occlusion, bile leak, peritonitis, and bleeding [56,57]. EUS-GBD demonstrates a low reintervention rate and a manageable safety profile even when used as rescue therapy after failed ERCP and EUS-CDS [58].

## 3. Altered Anatomy

Surgically altered anatomy (SAA) refers to patients who have undergone gastrointestinal reconstructive procedures such as Roux-en-Y gastric bypass (RYGB), Roux-en-Y hepaticojejunostomy, pancreaticoduodenectomy (Whipple), or Billroth II reconstruction [59]. These surgeries disrupt conventional endoscopic access to the ampulla and biliary tree, complicating management of biliopancreatic disorders. According to biliary drainage requirements, whether achieving anterograde or retrograde drainage for temporary relief, or establishing permanent biliary drainage by means of EUS-assisted techniques, different approaches may be considered. It is worth noting that some of these have been described in previous sections and are applicable to SAAs. In this context, t-EUS has become the key for minimally invasive interventions, allowing the introduction and implementation of EUS-guided transenteric ERCP (EDEE) and EUS-directed transgastric ERCP (EDGE). Other techniques, though not the object of this review, such as complementary use of enteroscopy, may also be applied in this context. The malignant or benign nature must always be considered when choosing the most appropriate approach, as more aggressive and permanent interventions (EUS-CDS/-HGS/-GBD) may be preferred in a malignant setting, whilst an anatomy-preserving approach in benign settings is desirable. This section will focus on EDEE and EDGE procedures.

EDGE involves creating a temporary fistula between the gastric pouch (or jejunum) and the excluded stomach using a LAMS, allowing a standard duodenoscope to access the duodenum and perform ERCP as in native anatomy. This approach is specifically suited for patients with RYGB anatomy. By contrast, EDEE creates a de novo enteroenteric anastomosis (e.g., jejunojejunostomy or gastrojejunostomy) under EUS guidance using a LAMS to access the biliary limb or hepaticojejunostomy, enabling ERCP in a broader range of SAAs, including Roux-en-Y hepaticojejunostomy or Whipple procedures.

EDEE has high clinical and technical success, being preferable in benign indications and when multiple ERCPs are anticipated [60]. Its limitations include a significant rate of AEs, including LAMS misplacement during the passage of the scope through the LAMS, technical complexity, and persistent fistula formation [60]. European guidelines indicate that EDGE may be considered in patients that have previously undergone RYGB over the use of other techniques, in particular EDEE, because of its high technical and clinical success rates and lower rates of AEs [16,61].

Both procedures are increasingly being performed in expert centers and future research is directed at multicenter prospective trials to validate efficacy and safety across diverse practice settings [61,62]. From a practical standpoint, the clinical question of which SAA patients should be referred for t-EUS-based access versus percutaneous or surgical alternatives requires careful multidisciplinary discussion. Comparative studies are needed to clarify long-term outcomes, optimal patient selection, and management of persistent fistula, especially for EDEE. Ongoing development of dedicated LAMS use during EDEE with anti-migratory features and thin delivery systems aims to reduce adverse events such as stent migration and persistent fistula formation.

## 4. Pancreatic Interventions

### 4.1. Pancreatic Fluid Collection

EUS-guided drainage of pancreatic fluid collections (PFCs) has become the standard of care for the management of symptomatic collections following acute pancreatitis or pancreatic surgery, replacing surgical and percutaneous interventions in a step-up approach [63]. Indications for drainage include persistent symptoms, sepsis, gastric or biliary obstruction, after the failure of conservative management [64]. Timing for EUS-guided drainage usually is postponed after antibiotic treatment [64] and typically requires a waiting time of four weeks; however, if patients’ conditions require anticipated drainage, recent data suggests the feasibility of early EUS drainage [65,66].

EUS-guided drainage is typically performed with a large LAMS (from 15 to 20 mm) through the gastric or duodenal wall, depending on the anatomical relationship between the collection and the lumen. The introduction of LAMSs has significantly simplified EUS-guided PFC drainage by allowing single-step access, effective drainage, and the possibility of direct endoscopic necrosectomy when required [63].

In cases of pancreatic pseudocyst, the fluid content may not require the larger diameter provided by a LAMS, and plastic stents could theoretically offer comparable effectiveness. However, several studies have demonstrated superior clinical efficacy and easier single-step access with LAMSs [67]. On the other hand, in cases of WON, the possibility to perform direct endoscopic necrosectomy confers a significant advantage to LAMSs in terms of efficacy, number of interventions and time to resolution.

An RCT by Bang et al. has demonstrated that endoscopic management of infected necrotizing pancreatitis is associated with technical success rates exceeding 90%, lower rates of adverse events, shorter hospital stay, and reduced healthcare costs compared with surgical approaches [68]. Some issues are still debated, especially the timing for necrosectomy: recent trials suggests that upfront necrosectomy could reduce hospitalization time and the number of necrosectomies, although improvement of overall survival is probably not affected [69,70]. One limitation of endoscopic necrosectomy is the use of devices not specifically designed for this purpose (e.g., snares, baskets, forceps); however, specific tools, such as Endo Rotor and Necrolith, have recently been introduced in the market to obviate this problem [71,72]. Despite its effectiveness, EUS-guided management of PFCs carries a non-negligible risk of complications, mainly related to intra-collection bleeding, and infection [64]. Placement of co-axial double pig-tail stent [73], prophylactic pseudoaneurysm embolizations, use of CO2 insufflation and proper antimicrobial treatment may aid in AE reduction [64].

### 4.2. Pancreatic Duct Drainage

EUS-guided pancreatic duct drainage (EUS-PD) represents one of the most technically challenging procedures among t-EUS interventions. It is primarily indicated in patients with obstructive chronic pancreatitis, post-surgical pancreaticojejunostomy strictures, pancreatic fistula or pancreatic duct disconnection syndrome following severe acute pancreatitis [74]. In general, EUS-PD is currently considered after failure of conventional or enteroscopy-assisted ERCP, or when these approaches are deemed technically unfeasible [75]. However, in selected cases such as SAA or duodenal obstruction, EUS-PD may be considered as a first-line option [76].

Contraindications can be divided into technical-related, which include a main pancreatic duct diameter too small or a long distance between the inlet site and the pancreas (e.g., in massive ascites), and patient-related, which include severe coagulopathy or unstable clinical conditions [74].

Two principal approaches of EUS-PD are currently employed: EUS-guided rendezvous (EUS-RV), previously discussed, and EUS-guided transmural pancreatic duct drainage/anastomosis (EUS-D/A) [75].

EUS-guided transmural drainage consists of the creation of a direct fistula between the pancreatic duct and the gastrointestinal lumen. This access can be used either for pushing a transpapillary stent (EUS-antegrade stenting, EUS-AG) or to create permanent anastomosis such as EUS-pancreaticogastrostomy (EUS-PGS), pancreatic duodenostomy or pancreaticojejunostomy, depending on patient’s anatomy [75]. EUS-PD is among the most technically demanding of all t-EUS procedures, and outcomes are strongly operator- and center-dependent. In practical terms, it should be considered only after thorough multidisciplinary evaluation confirming that conventional ERCP, enteroscopy-assisted ERCP, and percutaneous approaches are unavailable or have failed.

Following duct puncture, tract dilation is performed to allow placement of a plastic or metal stent between the pancreatic duct and the gastrointestinal lumen. The antegrade approach is generally performed as a two-step procedure, in which guidewire passage and stent placement across the papilla are obtained after fistula maturation [76,77]. In cases of pancreatic duct obstruction due to stone impaction, adjunctive therapies such as pancreatoscopy-guided lithotripsy may further enhance ductal clearance [78]. Peroral pancreatoscopy may also play a role in the etiological assessment of pancreatic duct strictures, potentially influencing subsequent management. In cases of benign strictures that resolve over time, the transmural stent placed during EUS-PGS can eventually be removed [75].

According to a systematic review by Nakai et al., transmural EUS-PD demonstrated higher technical success rates compared with the rendezvous approach, although the overall technical success remained modest [76]. The overall adverse event rate was 21.3%, highlighting the complexity and risk profile of the procedure. Reported complications include abdominal pain, post-procedural pancreatitis, pancreatic juice leakage, peritonitis, pancreatic fluid collection formation, stent migration, bleeding, and perforation [76]. These data emphasize the need for careful patient selection and performance of EUS-PD in high-volume expert centers.

## 5. EUS-Guided Gastroenteric Anastomosis in Pancreatic Cancer

Malignant gastric outlet obstruction (GOO) is a condition defined by the impaired passage of luminal content through the pyloric region due to the presence of a malignant obstruction, which can cause epigastric pain, early satiety, vomiting, weight loss and an overall reduction in the patients’ quality of life. Its etiology is usually linked to neoplasms of the stomach, duodenum or of the biliopancreatic tract, especially pancreatic cancer, where it can occur in about 10–25% of cases [79]. It is considered a negative prognostic factor as it is associated with a lower quality of life and higher morbidity, and it can undermine the tolerance to oncological treatment [80].

Traditionally, surgical gastrojejunostomy has been the treatment of choice for GOO management, but its application is limited by high perioperative morbidity and mortality, especially in patients with poor performance status and a shorter life expectancy. Endoscopic enteral stenting with self-expanding metallic stents (SEMSs) was proposed as a valid alternative for high surgical risk patients, thanks to its safety, wide availability and rapid ability to induce symptom remission; unfortunately, stent disfunction (migration, obstruction, failure) is common and the reintervention rate is high [81,82,83].

Therapeutic EUS offers a solution to GOO through a hybrid approach: EUS-guided gastroenterostomy (EUS-GE) with LAMS combines the advantages of stable anastomosis with the minimally invasive nature of endoscopy. Technical success, defined as the correct deployment and placement of the stent, was demonstrated to be high [84,85], while the AE rate can vary from 0 to 26% [24,86], with the most common ones being stent misdeployment, enteric leakage with peritonitis, and bleeding. The progressive standardization of the technique [87], especially with the free-hand approach (WEST techniques) allowed reduction of LAMS misdeployment [88,89]. Ascites is apparently the only negative predictive factor, although not an absolute contraindication to the procedure, while the distance between the lumen was demonstrated to be the most relevant predictor of technical success [90,91].

**Figure 2:**
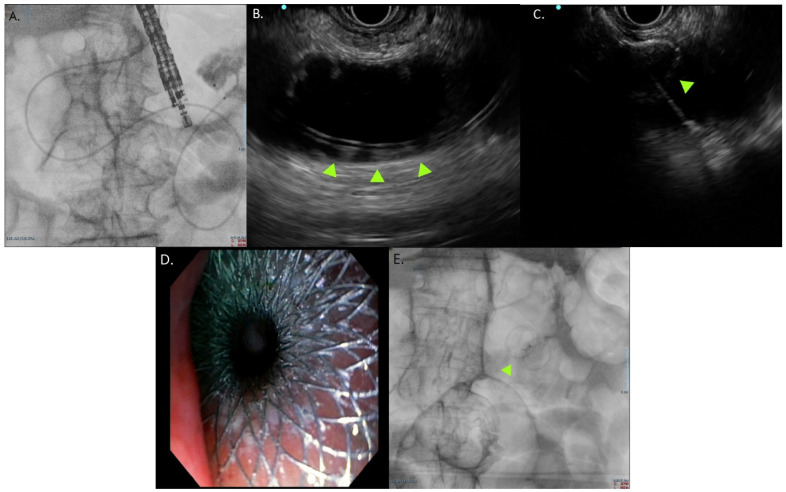
Gastroenterostomy steps. (**A**) Jejunal loop is distended with contrast medium and saline solution after the insertion of a nasojejunal tube. (**B**) Ultrasound view of the distended jejunal loop. The green arrows show the nasojejunal tube within the loop lumen. (**C**) Ultrasound view of the opening of the distal flange of the LAMS (green arrow) within the jejunal loop lumen. (**D**) Endoscopic view of the proximal flange of the LAMS in the gastric lumen. Through the stent, the jejunal mucosa can be visualized. (**E**) Final fluoroscopic view of the LAMS (green arrow).

Recent studies compared EUS-GE with surgical gastrojejunostomy (SGJ) demonstrated that EUS-GE achieves comparable or superior clinical success to SGJ, with fewer AEs, shorter time to oral intake, and reduced hospital stay [92,93]. EUS-GE offers a valid, lasting and safe option for the treatment of malignant GOO and many experts agree in considering it the first choice when available [16,94]. Currently, the challenging learning curve and the limited availability of expertise still hinder its large-scale application. Figure 2 illustrates the steps undertaken in EUS-GE.

## 6. EUS-Guided Pancreatic Lesion Locoregional Treatments

EUS-RFA is a novel and promising approach for the local treatment of pancreatic lesions. Traditionally, the gold standard treatment for pancreatic cancer, as well as for pancreatic neuroendocrine tumors (pNETs) and pre-malignant lesions, has been surgical resection; however, pancreatic surgery is not always feasible due to technical and/or clinical reasons and is often associated with post-operative morbidity and mortality [95]. Thus, the need for a less invasive approach to pancreatic lesions has been growing and EUS-RFA has established a role for locoregional treatment.

RFA involves the delivery of a high-frequency (400–500 Hz) alternating current in order to induce thermal damage to the target tissue, inducing coagulative necrosis and, eventually, apoptosis of the target cells [96].

### 6.1. Pancreatic Neuroendocrine Tumors

The most established application of EUS-RFA is local treatment of pancreatic neuroendocrine tumors (PNETs). These are rare neoplasms, accounting for approximately 30% of all GI NETs.

The European Neuroendocrine Tumor Society (ENETS) considers surgical resection the treatment of choice for most PNETs, irrespective of their functionality; EUS-RFA has recently been approved in ENETS guidelines for insulinomas ≤ 2 cm in patients unfit for surgery in experienced centers [97,98]. In this specific setting, multiple studies confirmed not only the feasibility, but also the efficacy of EUS-RFA, which was considered comparable to surgery, at least in controlling neuroendocrine symptoms, but with a lower rate of adverse events and a shorter hospital stay [99,100,101,102,103,104]. Napoléon et al. confirmed the acceptable safety of the procedure, with adverse events (AEs) reported in 19% of patients and the majority of them being epigastric pain and mild acute pancreatitis; the only statistically significant risk factor for adverse events was the proximity ≤1 mm) of the neoplasm to the main pancreatic duct [105].

Regarding nonfunctioning-PNETs, data are still scarce and no clear indication has been made. ENETS guidelines recommends surgical resection for lesions > 2 cm, while surveillance should be the approach of choice for asymptomatic masses ≤ 2 cm, with a Ki-67 index < 5% (G1 and low G2 NETs) and no signs of malignancy on an abdominal CT scan and 68-Ga-DOTATATE PET-CT [98]. The two biggest case-series available [104,105] showed promising results in both efficacy and safety: both used EUS-RFA to treat NF-PNETs ≤ 2 cm and showed a complete response in about 71% of cases, with just one serious AE (MPD stenosis) reported after treating a lesion with a distance from the MPD < 1 mm.

### 6.2. Pancreatic Adenocarcinoma and Pancreatic Metastases

RFA offers a novel inviting approach for local ablation of pancreatic ductal adenocarcinoma (PDAC), aiming at reducing symptoms, improving survival and even allowing a downstaging for borderline resectable cancers. A study by Song et al. studied the feasibility and safety of the procedure on six patients, with just two patients experiencing post-procedure mild abdominal pain treated with analgesics [106]. Further studies confirmed the safety of this technique and assessed the radiological response as well [107,108,109], though data on long-term outcomes are still lacking; a median overall survival of 20 to 24 months was suggested in recent prospective studies [110,111].

EUS-RFA may also have a systemic immunomodulatory effect known as the abscopal effect: through tissue damage, RFA induces the release of tumor-related antigens, thought to promote not only local microenvironment remodeling, but also a distant immune response that could be exploited to amplify the response to oncological treatment [112,113]. For example, a novel and promising application lies in the delivery of active microparticles directly into pancreatic cancer lesions as suggested by Lim et al. [114].

Crinò et al. first described the ablation of a pancreatic head metastasis from renal cell carcinoma (RCC) with an EUSRA needle, obtaining a necrosis of 73.5% of the tumor volume, with a decent safety profile [108]. Similar results were obtained by Biasutto et al. [115] on a four-patient cohort, three of which were treated with EUS-RFA. The biggest study available described 21 pancreatic RCC metastases treated with 26 sessions of EUS-RFA, reporting two severe AEs and no deaths [116]. This technique seems feasible and safe in managing pancreatic metastasis, but long-term data are still missing.

### 6.3. Pancreatic Cystic Lesions

Application of RFA in pancreatic cystic lesions (PCLs) is limited to cysts that carry a high risk of malignant evolution, typically intraductal papillary mucinous neoplasms (IPMNs) with worrisome features or high-risk stigmata or mucinous cystic neoplasms (MCNs) [117].

The first experience with EUS-RFA on PCLs was reported by Pai et al. [118] who treated a total of six patients (four MCNs, one IPMN and one microcystic adenoma) showing a reduction of about 50% of the tumor volume, without significant adverse events. Corroborating results were then published by Barthet et al. [119] who ablated 16 IPMNs with worrisome features and one MCN, obtaining an overall satisfying result over short- and long term. On the other hand, Oh et al. [120] reported mediocre success after ablating 13 SCNs, with five patients not reaching a reduction > 30% in lesion diameter, which was established as the resolution goal.

**Figure 3:**
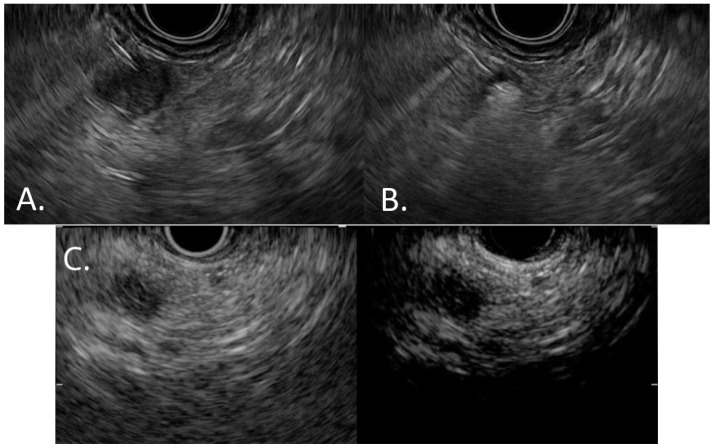
EUS-RFA of a pancreatic lesion. (**A**) EUS-guided insertion of the RFA needle into the lesion. (**B**) Ablation of neoplastic tissue is obtained as shown by the hyperechoic area inside the lesion. (**C**) Contrast-enhanced, post-ablation EUS showing a complete anechoic area in the site of the lesion, without any contrast enhancement, that confirms the success of the treatment.

Yet again, EUS-RFA seems a valid and safe approach in inducing a volumetric reduction and managing symptomatic PCLs, but it is still debated if it has any effect on controlling the malignant potential of these lesions and its implication in long-term survival. Figure 3 illustrates how EUS-RFA is applied to pancreatic lesions.

### 6.4. Celiac Plexus Neurolysis

Managing pain in patients with advanced pancreatic cancer is essential in a palliative care setting and the neurolysis of the celiac plexus (CPN) is the technique proposed to reach this goal.

Jin et al. [121] first successfully applied EUS-RFA to induce CPN in a 57-year-old man with metastatic pancreatic cancer; after the procedure, the visual analog pain score decreased from 8 to 2, without any AE reported. Bang et al. [122] later performed a single-blind, randomized trial comparing EUS-CPN with dehydrated alcohol and EUS-RFA, showing not only feasibility and safety of EUS-RFA, but also better pain control compared to alcoholization.

## 7. Conclusions

The application of t-EUS in biliopancreatic diseases has evolved from a niche rescue approach to a cornerstone of multidisciplinary management across a wide spectrum of conditions. Moreover, the standardization of the different techniques and the progressive diffusion to low-volume or non-academic centers has also allowed the implementation of t-EUS in clinical practice. Its benefits are conditional on careful patient selection, adequate institutional infrastructure, and honest appraisal of the evidence.

## Data Availability

No new data were created or analyzed in this study.
